# Supplementary material for: Curated and harmonised transcriptomics datasets of interstitial lung diseases
Source: Data Brief. 2025 Oct 14;63:112139. doi: 10.1016/j.dib.2025.112139 (PMC12581653; doi:10.1016/j.dib.2025.112139)
Supplement: Supplementary file 1 [file mmc1.zip › Supplementary_material/RNA-seq/fastQC reports/GSE138283/SRR10213001_fastqc.html]

SRR10213001.fastq.gz FastQC Report 

FastQC Report

Fri 2 Feb 2024  
SRR10213001.fastq.gz

## Summary

- Basic Statistics
- Per base sequence quality
- Per sequence quality scores
- Per base sequence content
- Per sequence GC content
- Per base N content
- Sequence Length Distribution
- Sequence Duplication Levels
- Overrepresented sequences
- Adapter Content

## Basic Statistics

| Measure | Value |
| --- | --- |
| Filename | SRR10213001.fastq.gz |
| File type | Conventional base calls |
| Encoding | Sanger / Illumina 1.9 |
| Total Sequences | 93600373 |
| Sequences flagged as poor quality | 0 |
| Sequence length | 35-76 |
| %GC | 48 |

## Per base sequence quality

## Per sequence quality scores

## Per base sequence content

## Per sequence GC content

## Per base N content

## Sequence Length Distribution

## Sequence Duplication Levels

## Overrepresented sequences

| Sequence | Count | Percentage | Possible Source |
| --- | --- | --- | --- |
| CCACAAATTATGCAGTCGAGTTTCCCACATTTGGGGAAATCGCAGGGGTCAGCACATCCGGAGTGCAATGGATA | 885502 | 0.9460453752678957 | No Hit |
| CCCCACTACCACAAATTATGCAGTCGAGTTTCCCACATTTGGGGAAATCGCAGGGGTCAGCACATCCGGAGTGCA | 824142 | 0.8804900809529894 | No Hit |
| CCCACTACCACAAATTATGCAGTCGAGTTTCCCACATTTGGGGAAATCGC | 579304 | 0.6189120635235076 | No Hit |
| CACAAATTATGCAGTCGAGTTTCCCACATTTGGGGAAATCGCAGGGGTCA | 506208 | 0.5408183576362458 | No Hit |
| CTCAGACCGCGTTCTCTCCCTCTCACTCCCCAATACGGAGAGAAGAACGATCATCAATGGCTGACGGCAGTTGC | 343313 | 0.3667859315047815 | No Hit |
| GTCGAGTTTCCCACATTTGGGGAAATCGCAGGGGTCAGCACATCCGGAGTGCAATGGATAAGCCTCGCCCTGGGA | 322982 | 0.345064864218009 | No Hit |
| CTCCGTTTCCGACCTGGGCCGGTTCACCCCTCCTTAGGCAACCTGGTGGT | 263089 | 0.28107687134964726 | No Hit |
| CCTTAGGCAACCTGGTGGTCCCCCGCTCCCGGGAGGTCACCATATTGATG | 214276 | 0.22892643814571126 | No Hit |
| GTCCCCCACTACCACAAATTATGCAGTCGAGTTTCCCACATTTGGGGAAA | 200834 | 0.21456538426401356 | No Hit |
| CTACCACAAATTATGCAGTCGAGTTTCCCACATTTGGGGAAATCGCAGGG | 190232 | 0.20323850632518312 | No Hit |
| CTGGAGTCTTGGAAGCTTGACTACCCTACGTTCTCCTACAAATGGACCTTGAGAGCTTGTTTGGAGGTTCTAGC | 180941 | 0.19331226383040162 | No Hit |
| CCCCTCCTTAGGCAACCTGGTGGTCCCCCGCTCCCGGGAGGTCACCATAT | 180047 | 0.19235713943148497 | No Hit |
| CCAGGCTGGAGTGCAGTGGCTATTCACAGGCGCGATCCCACTACTGATCA | 167670 | 0.1791339015283625 | No Hit |
| CACTACCACAAATTATGCAGTCGAGTTTCCCACATTTGGGGAAATCGCAG | 162025 | 0.17310294265600845 | No Hit |
| CTCAGGCTGGAGTGCAGTGGCTATTCACAGGCGCGATCCCACTACTGATC | 157050 | 0.16778779289693643 | No Hit |
| CCACTACCACAAATTATGCAGTCGAGTTTCCCACATTTGGGGAAATCGCA | 155194 | 0.16580489481596403 | No Hit |
| CCCTCCTTAGGCAACCTGGTGGTCCCCCGCTCCCGGGAGGTCACCATATT | 140597 | 0.15020987149271295 | No Hit |
| GTCTGGAGTCTTGGAAGCTTGACTACCCTACGTTCTCCTACAAATGGACC | 139073 | 0.14858167285294901 | No Hit |
| GTGGCTATTCACAGGCGCGATCCCACTACTGATCAGCACGGGAGTTTTGA | 136220 | 0.14553360807654045 | No Hit |
| ACCACAAATTATGCAGTCGAGTTTCCCACATTTGGGGAAATCGCAGGGGTCAGCACATCCGGAGTGCAATGGATA | 134861 | 0.14408169078556984 | No Hit |
| CCCCCACTACCACAAATTATGCAGTCGAGTTTCCCACATTTGGGGAAATCGCAGGGGTCAGCACATCCGGAGTGC | 111330 | 0.1189418337040174 | No Hit |
| CAGGCTGGAGTGCAGTGGCTATTCACAGGCGCGATCCCACTACTGATCAG | 102786 | 0.10981366495195484 | No Hit |
| GTCGATGCGTGGAGTGGACGGAGCAAGCTCCTATTCCATCTCCTATTTCC | 99386 | 0.1061812007950011 | No Hit |
| GCTCAGGCTGGAGTGCAGTGGCTATTCACAGGCGCGATCCCACTACTGAT | 99267 | 0.10605406454950773 | No Hit |
| ATGCAGTCGAGTTTCCCACATTTGGGGAAATCGCAGGGGTCAGCACATCC | 95337 | 0.10185536333279356 | No Hit |
| CTCCTTAGGCAACCTGGTGGTCCCCCGCTCCCGGGAGGTCACCATATTGA | 93839 | 0.10025494236011218 | No Hit |

## Adapter Content

Produced by FastQC (version 0.11.7)
